# Frequent Transposition of Multiple Insertion Sequences in *Geobacillus kaustophilus* HTA426

**DOI:** 10.3389/fmicb.2021.650461

**Published:** 2021-03-24

**Authors:** Hirokazu Suzuki, Tatsunari Taketani, Misaki Tanabiki, Misaki Ohara, Jyumpei Kobayashi, Takashi Ohshiro

**Affiliations:** ^1^Faculty of Engineering, Tottori University, Tottori, Japan; ^2^Center for Research on Green Sustainable Chemistry, Tottori University, Tottori, Japan; ^3^Department of Engineering, Graduate School of Sustainability Science, Tottori University, Tottori, Japan; ^4^Department of Chemistry and Biotechnology, Graduate School of Engineering, Tottori University, Tottori, Japan

**Keywords:** extra-cytoplasmic function sigma factor, IS*4*, IS*701*, IS*1634*, IS*Lre2*, stress-induced transposition, thermophile, transposable element

## Abstract

*Geobacillus kaustophilus* HTA426 is a thermophilic bacterium whose genome harbors numerous insertion sequences (IS). This study was initially conducted to generate mutant genes for thermostable T7 RNA polymerase in *G. kaustophilus*; however, relevant experiments unexpectedly identified that the organism transposed multiple IS elements and produced derivative cells that expressed a silent gene via transposition. The transposed elements were diverse and included members of the IS*4*, IS*701*, IS*1634*, and IS*Lre2* families. The transposition was relatively active at elevated temperatures and generated 4–9 bp of direct repeats at insertion sites. Transposition was more frequent in proliferative cells than in stationary cells but was comparable between both cells when *sigX*, which encodes an extra-cytoplasmic function sigma factor, was forcibly expressed. Southern blot analysis indicated that IS transposition occurred under growth inhibitory conditions by diverse stressors; however, IS transposition was not detected in cells that were cultured under growth non-inhibitory conditions. These observations suggest that *G. kaustophilus* enhances IS transposition via *sigX*-dependent stress responses when proliferative cells were prevented from active propagation. Considering *Geobacillus* spp. are highly adaptive bacteria that are remarkably distributed in diverse niches, it is possible that these organisms employ IS transposition for environmental adaptation via genetic diversification. Thus, this study provides new insights into adaptation strategies of *Geobacillus* spp. along with implications for strong codependence between mobile genetic elements and highly adaptive bacteria for stable persistence and evolutionary diversification, respectively. This is also the first report to reveal active IS elements at elevated temperatures in thermophiles and to suggest a sigma factor that governs IS transposition.

## Introduction

Insertion sequences (IS) are a simple class of mobile genetic elements that propagate themselves or change the position in the host’s genetic material via replicative or non-replicative transposition, respectively (Vandecraen et al., 2018). An IS element is flanked by short inverted repeats and encodes a transposase that catalyzes transposition. In addition, the IS element may carry regulatory genes essential for transposition but does not carry accessory genes. A total of 32 IS families are classified, and most encode DDE-type (named for a conserved amino acid triad) transposases (Siguier et al., 2015). Transposition potentially causes deleterious mutations; therefore, IS elements were initially considered parasitic and selfish factors that multiply without conferring a survival advantage to the host organism (Schrader and Schmitz, 2019). However, it is now known that IS transposition can provide evolutionary adaptation for their hosts via gene inactivation and/or modulated expression of the neighboring genes (Vandecraen et al., 2018).

Insertion sequences that employ DDE-type transposases can achieve either replicative transposition or non-replicative transposition (Bouuaert and Chalmers, 2010). Non-replicative transposition results from cut-and-paste mechanisms where the transposase expressed from an IS element excises the IS from the original site and integrates it into another site. Replicative transposition is performed by either copy-and-paste (donor-primed replication) or copy-in (target-primed replication) mechanisms. In the former mechanism, the transposase releases a single-stranded and circular IS element from the original site. The IS element undergoes replication and eventually integrates into another site. The latter mechanism uses transposases to nick IS termini and directly ligate them with another site. This results in formation of a Shapiro intermediate followed by a cointegrate molecule via DNA replication. Subsequently, the intermediate undergoes recombination to generate the final products. All these mechanisms integrate IS elements into target sites while generating gaps. The gaps are then filled by the DNA repair systems of the host organisms; therefore, IS elements are generally flanked by direct repeats (DR).

*Geobacillus* spp. are gram-positive thermophiles that can form endospores. The species rapidly grow at >55°C but are unusually distributed in diverse habitats including cool or ambient environments (Suzuki, 2018). This is partially explained by the distribution of their endospores (Zeigler, 2014). However, it is equally possible that *Geobacillus* spp. grow as proliferative cells in numerous environments because they often exhibit unique capacities to reproduce in the respective habitats (Suzuki, 2018). Pan-genomic analysis suggests that *Geobacillus* spp. have remarkably diversified their genomes (Bezuidt et al., 2016). *Geobacillus* spp. can also rapidly generate mutant genes for thermostable variants from thermolabile protein genes (Liao et al., 1986; Suzuki et al., 2015; Kobayashi et al.,2015a,b; Wada et al., 2016). In the species, mutagenesis is apparently induced when proliferative cells are faced with growth inhibition by antibiotics (Suzuki et al., 2018). These observations suggest that *Geobacillus* spp. are active in environmental adaptation via genetic diversification.

We have studied generation and selection of thermostable proteins in *Geobacillus kaustophilus* HTA426 (Suzuki et al., 2015; Kobayashi et al.,2015a,b; Wada et al., 2016). This strain originates from a mud sample of the Mariana Trench and can grow between 42°C and 74°C (Takami et al., 2004a). In related studies, we unexpectedly discovered that multiple IS elements could perform transposition in this organism. Mobile genetic elements in thermophiles are insufficiently characterized; therefore, we analyzed this phenomenon in detail. Here, we report that *G. kaustophilus* performs frequent and genome-wide IS transposition potentially via *sigX*-dependent stress responses when proliferative cells were prevented from active propagation.

## Materials and Methods

### Genetic Tools

Plasmid pGAM46 was previously constructed for marker-free gene integration into the *amyA* gene in *G. kaustophilus* (Suzuki et al., 2012). Plasmid pGKE75 (Kobayashi et al., 2015a) was used for forcible gene expression under the control of the gk704 promoter (P_*gk*__704_) of *G. kaustophilus* (Suzuki et al., 2013b). Plasmid pGKE74 was constructed from pGKE75 via elimination of the P_*gk*__704_ region. Plasmids were introduced into *G. kaustophilus* using conjugative plasmid transfer from *Escherichia coli* (Suzuki et al., 2013a). Chromosomal gene replacement was performed using the procedure previously described (Suzuki et al., 2012). The primer sequences are summarized in the Supplementary Table 1.

### Construction of Plasmids

Table 1 summarizes relevant plasmids. T7 promoter (P_*T*__7_) was amplified from pET-16b (Merck KGaA, Darmstadt, Germany) using the primers t7_–__250__*F*_ and t7_0__*R*_. The *pyrF* gene encoding for orotidine 5′-phosphate decarboxylase of *G. kaustophilus* was amplified using the primers pyrF_0__*F*_ and pyrF_*TR*_. The P_*T*__7_ and *pyrF* fragments were cloned between the *Hin*dIII and *Sph*I sites and the *Sph*I and *Bam*HI sites, respectively, of pGAM46 to give pGAM46P_*T*__7_-*pyrF* carrying the P_*T*__7_-*pyrF* cassette. The gene for T7 RNA polymerase (T7RP) was amplified from *E. coli* BL21(DE3) using the primers t7RP_0__*F*_ and t7RP_*TR*_ and cloned between the *Sph*I and *Bam*HI sites of pGKE75 to give pGKE75-*T7RP*, which carried the P_*gk*__704_-*T7RP* cassette. To construct the P_*gk*__704_-*sigB* cassette, P_*gk*__704_ was amplified from pGKE75 using the primers gk704_–__250__*F*_ and sigB_0__*R*_; *sigB* encoding for sigma factor B (SigB) of *G. kaustophilus* was amplified using the primers sigB_0__*F*_ and sigB_*TR*_. These fragments were combined using fusion PCR and cloned between the *Sph*I and *Bam*HI sites to give pGKE74P_*gk*__704_-*sigB*. To construct the P_*gk*__704_-*rsbV* cassette, P_*gk*__704_ was amplified using the primers gk704_–__250__*F*_ and rsbV_0__*R*_; *rsbV* encoding for anti-SigB antagonist (RsbV) of *G. kaustophilus* was amplified using the primers rsbV_0__*F*_ and rsbV_*TR*_. These fragments were combined and cloned between the *Hin*dIII and *Bam*HI sites of pGKE74 to give pGKE74P_*gk*__704_-*rsbV*. To construct the P_*gk*__704_-*sigX* cassette, P_*gk*__704_ was amplified using the primers gk704_–__250__*F*_ and sigX_0__*R*_; *sigX* encoding for sigma factor X (SigX) of *G. kaustophilus* was amplified using the primers sigX_0__*F*_ and sigX_*TR*_. These fragments were combined and cloned between the *Hin*dIII and *Bam*HI sites to give pGKE74P_*gk*__704_-*sigX*.

**TABLE 1:** *Geobacillus kaustophilus* strains and plasmids used in this study.

Strain or plasmid	Relevant description	References
**Strain**		
HTA426	Wild-type strain	Takami et al., 2004b
MK242	Control strain derived from HTA426; Δ*pyrF* Δ*pyrR* Δ*hsdM_1_S_1_R_1_* Δ(*mcrB*_1_-*mcrB*_2_-*hsdM_2_S_2_R_2_*-*mrr*) *amyA*::P_*gk*__704_-*bgaB*	Suzuki et al., 2015
MK480	Error-prone strain derived from MK242; Δ*mutSL* Δ*mutY* Δ*ung* Δ*mfd*	Suzuki et al., 2015
MK534	Error-prone strain derived from MK480; *amyA*::P_*T*__7_-*pyrF*	This study
MK534_*T*__7__*RP*_	Error-prone strain derived from MK534; pGKE75-*T7RP*	This study
MK536	Control strain derived from MK242; *amyA*::P_*T*__7_-*pyrF*	This study
MK536_*up*__1_	Uracil prototroph derived from MK536; IS*Gka1*-*pyrF*	This study
MK536_*p*__74_	Control strain derived from MK536; pGKE74	This study
MK536_*rsbV*_	*rsbV* expressor derived from MK536; pGKE74P_*gk*__704_-*rsbV*	This study
MK536_*sigB*_	*sigB* expressor derived from MK536; pGKE74P_*gk*__704_-*sigB*	This study
MK536_*sigX*_	*sigX* expressor derived from MK536; pGKE74P_*gk*__704_-*sigX*	This study
**Plasmid**		
pGAM46	Integration vector for *G. kaustophilus*	Suzuki et al., 2012
pGAM46P_*T*__7_-*pyrF*	pGAM46 derivative used to integrate P_*T*__7_-*pyrF* cassette at GK0707 locus	This study
pGKE75	Expression vector carrying P_*gk*__704_ for *G. kaustophilus*	Kobayashi et al., 2015a
pGKE75-*T7RP*	pGKE75 derivative used for forcible *T7RP* expression	This study
pGKE74	pGKE75 derivative without P_*gk*__704_	This study
pGKE74P_*gk*__704_-*rsbV*	pGKE74 derivative carrying P_*gk*__704_-*rsbV* cassette for *rsbV* expression	This study
pGKE74P_*gk*__704_-*sigB*	pGKE74 derivative carrying P_*gk*__704_-*sigB* cassette for *sigB* expression	This study
pGKE74P_*gk*__704_-*sigX*	pGKE74 derivative carrying P_*gk*__704_-*sigX* cassette for *sigX* expression	This study

*The T7 and gk704 promoters are abbreviated as P_*T*__7_ and P_*gk*__704_, respectively. The *pyrF* gene encodes for a pyrimidine biosynthetic enzyme. Plasmids pGKE74P_*gk*__704_-*rsbV*, pGKE74P_*gk*__704_-*sigB*, pGKE74P_*gk*__704_-*sigX*, and pGKE75-*T7RP* direct production of anti-sigma factor B antagonist (RsbV), sigma factor B (SigB), sigma factor X (SigX), and T7 RNA polymerase (T7RP), respectively, under the P_*gk*__704_ control in *G. kaustophilus*. These plasmids carry kanamycin-resistant gene for thermophiles. Most strains lack genes for pyrimidine biosynthesis (*pyrF* and *pyrR*) and restriction–modification systems (*hsdM_1_S_1_R_1_*, *mcrB*_1_, *mcrB*_2_, *hsdM_2_S_2_R_2_*, and *mrr*). Error-prone strains also lack genes for DNA repair (*mutS*, *mutL*, *mutY*, *ung*, and *mfd*).*

### Bacterial Strains

Table 1 summarizes thermophilic strains used in this study. *G. kaustophilus* strains MK242 and MK480 were previously constructed from *G. kaustophilus* HTA426 (Suzuki et al., 2015). The P_*gk*__704_-*bgaB* cassette in MK242 and MK480 was replaced with the P_*T*__7_-*pyrF* cassette using pGAM46P_*T*__7_-*pyrF* to give *G. kaustophilus* strains MK536 and MK534, respectively. *G. kaustophilus* MK534 was transformed with pGKE75-*T7RP* to provide *G. kaustophilus* MK534_*T*__7__*RP*_. *G. kaustophilus* MK536 was transformed with pGKE74, pGKE74P_*gk*__704_-*rsbV*, pGKE74P_*gk*__704_-*sigB*, and pGKE74P_*gk*__704_-*sigX* to generate strains MK536_*p*__74_, MK536_*rsbV*_, MK536_*sigB*_, and MK536_*sigX*_, respectively. *G. kaustophilus* MK536_*up*__1_ was derived from *G. kaustophilus* MK536 via intrinsic IS transposition.

### Culture Conditions

*Geobacillus kaustophilus* was cultured in Luria–Bertani (LB; Nacalai Tesque, Kyoto, Japan) or semisynthetic (MM, MU, MC, or MN) media. The MM medium contained inorganic salts (K_2_SO4, 0.3 g/L; Na_2_HPO_4_⋅12H_2_O, 2.5 g/L; NH_4_Cl, 1 g/L; MgSO_4_, 0.4 g/L; MnCl_2_⋅4H_2_O, 3 mg/L; CaCl_2_⋅2H_2_O, 5 mg/L; and FeCl_3_⋅6H_2_O, 7 mg/L), 0.1% trace element solution (Amartey et al., 1991), Tris–HCl (10 mM, pH 7.5), uracil (10 mg/L), casamino acids (1 g/L), and D-glucose (10 g/L). The other semisynthetic media were based on MM medium; however, MU medium lacked uracil. The MC medium lacked both casamino acids and D-glucose, and MN lacked casamino acids and NH_4_Cl. Solid media contained agar (20 g/L). Kanamycin (5 mg/L) was added when necessary. The optical density at 600 nm (OD_600_) was monitored using an infrared-dependent detector (OD-Monitor A; Taitec, Saitama, Japan).

### Generation Assay of Uracil Prototrophs From *G. kaustophilus* MK536

*Geobacillus kaustophilus* MK536 was precultured overnight at 60°C in LB medium (5 mL). The cells were collected by centrifugation (14,000 × *g*, 10 s) and suspended in sterile water (1 mL) to remove medium elements. Cells were collected again by centrifugation and resuspended in sterile water (0.15 mL). The suspension was used to prepare a dilution series in sterile water, which was plated on MU media and then incubated at 65°C for 72 h to obtain uracil prototrophs. The dilution series was also incubated on MM plates for 24 h to determine viable cell concentrations. The generation frequency of uracil prototrophs was defined as the ratio of generated uracil prototrophs to incubated viable cells (10^5^–10^6^ cfu).

### Isolation of Genomic DNA

Mixtures were vigorously agitated during each addition of reagents. *G. kaustophilus* was cultured at 60°C in LB medium (30 mL). Cells were collected by centrifugation (4,400 × *g*, 5 min) and suspended in TEG buffer (3 mL) that contained Tris–HCl (25 mM, pH 8.0), ethylenediaminetetraacetic acid (10 mM), D-glucose (50 mM), lysozyme (1 mg/mL), and ribonuclease A (1 μg/mL). Following incubation at 37°C for 30 min, the suspension was mixed with sodium dodecyl sulfate (10%, 0.3 mL) and proteinase K (4 μg) and then incubated at 60°C for 30 min. The homogenate was supplemented with NaCl (5 M, 0.3 mL), cetyltrimethylammonium bromide (5%, 0.3 mL), and phenol/chloroform/isoamyl alcohol (25:24:1, 0.3 mL). After centrifugation (4,400 × *g*, 10 min), the aqueous supernatant was transferred to a conical tube and mixed with an equal volume of ethanol. The tube was repeatedly inverted to precipitate genomic DNA, which was washed twice with ethanol (70%, 1 mL) and dissolved in TE buffer (1 mL) that contained Tris–HCl (10 mM, pH 7.5) and ethylenediaminetetraacetic acid (1 mM). For next-generation sequencing, genomic DNA was further purified using a NucleoSpin gDNA Clean-up (Takara Bio, Otsu, Japan).

### Sequencing Analysis

The *pyrF* upstream region in uracil prototrophs was amplified using the primers pyrF_200__*R*_ and amyA_1300__*R*_. The amplicons were purified using the GenElute Agarose Spin Columns (Sigma Aldrich, St. Louis, MO, United States) and sequenced with Applied Biosystems 3730*xl* DNA Analyzer (Thermo Fisher Scientific, Waltham, MA, United States). Cycle sequencing reactions were performed using the BigDye Terminator v3.1 Cycle Sequencing Kit (Thermo Fisher Scientific) with the primers amyA_200__*F*_, amyA_400__*F*_, amyA_400__*R*_, amyA_800__*R*_, amyA_1300__*R*_, and/or pyrF_20__*R*_. The library for next-generation sequencing was constructed using an NEBNext Ultra DNA Library Prep Kit for Illumina (New England BioLabs, Ipswich, MA, United States) and validated using an Agilent 2100 Bioanalyzer (Agilent Technologies, Palo Alto, CA, United States). Sequences were obtained as 150 bp pair-end reads on a NovaSeq 6000 system (Illumina, San Diego, CA, United States). The assembly was first performed using Velvet (Zerbino and Birney, 2008) with default parameters. Based on the assembly, sequencing reads were aligned and assembled into contigs using SSPACE (Boetzer et al., 2011) and GapFiller (Boetzer and Pirovano, 2012). The draft sequence was compared with the complete sequence of *G. kaustophilus* HTA426 (Takami et al., 2004b) using BLAST^1^ to identify IS elements that had transposed. The read sequences were also mapped to the genome sequence of *G. kaustophilus* HTA426 using Burrows–Wheeler Aligner (Li and Durbin, 2010) and analyzed using Integrative Genomics Viewer (Thorvaldsdóttir et al., 2013) to confirm the IS transposition. The IS elements transposed in *G. kaustophilus* MK536_*up*__1_ were amplified using the following primers: is25F and is25R (at GK3299 locus); is28F and is28R (between GK1097 and GK1098 loci); is72F and is72R (at GK0885 locus); and is87F and is87R (between GK0301 and GK0302 loci). Amplicons were sequenced to determine the intact sequences.

### Transcription Analysis

*Geobacillus kaustophilus* strains MK536 and MK536_*up*__1_ were cultured in MM medium at 60°C. Cells were collected at OD_600_ = 1, and RNA was purified using an RNAprotect Bacteria Reagent and RNeasy Mini Kit (Qiagen, Venlo, Netherlands) with gDNA Eraser (Takara Bio). The *pyrF* transcript was detected using endpoint reverse transcription-polymerase chain reaction (RT-PCR). The RT reaction was performed using a PrimeScript RT reagent Kit (Takara Bio) with the PyrF_*TR*_ primer, whereas PCR was performed using Quick Taq HS DyeMix (Toyobo, Osaka, Japan) with two sets of primers: pyrF_0__*F*_ and pyrF_600__*R*_ (primer A) and is701_250__*F*_ and pyrF_200__*R*_ (primer B). Thermal cycles comprised 94°C for 2 min followed by 35 cycles of 94°C for 30 s, 55°C for 30 s, and 68°C for 2 min. The reaction without reverse transcriptase was used as negative control. The transcription of *rpoB*, which encodes for RNA polymerase β subunit, was detected as positive control using the primers rpoB_2800__*F*_ and rpoB_3800__*R*_.

### Southern Blot Analysis of IS*Gka1*/IS*Gka2* Transposition

*Geobacillus kaustophilus* MK536 was precultured in LB medium (5 mL) at 60°C. The cells were washed with sterile water (see above), and an aliquot (10^5^–10^6^ cfu) was incubated at 65°C on MU plates for 72 h. Generated colonies were purified on MU plates and used as uracil prototrophs, whereas background cells (without colony formation) were purified on LB plates and used as uracil auxotrophs. Washed cells (10^5^–10^6^ cfu) were also incubated at 65°C in liquid MU (20 mL) for 48 h. Cells were recovered on LB plates and screened using MM and MU plates to distinguish between uracil prototrophs and auxotrophs. In addition, washed cells were incubated at 65°C for as long as possible using media that prevent active cell growth: MC and MN media and LB medium supplemented with kanamycin (5 mg/L) or chloramphenicol (10 mg/L). Cells were recovered on LB plates from the resultant cultures. For successive culture under growth non-inhibitory conditions, MK536 was inoculated in LB medium (100 mL) and incubated at 65°C for 24 h. An aliquot of the culture (1 mL) was inoculated in fresh medium and further incubated under the same conditions. This process was repeated an additional five times. Subsequently, cells were colonized on LB plates. Respective clones were analyzed by Southern blotting to detect IS elements of *G. kaustophilus* (IS*Gka1* and IS*Gka2*). Genomic DNA (25 μg) was digested with *Dra*I and *Mun*I. The products were separated on an agarose gel (0.9%) by electrophoresis and transferred onto a nylon membrane to hybridize with a digoxigenin-labeled DNA probe. The probe was synthesized using a PCR DIG Probe Synthesis Kit (Roche, Basel, Switzerland) with the primers is701_250__*F*_ and is701_800__*R*_. Hybridized DNA was detected using a DIG Nucleic Acid Detection Kit (Roche).

### Generation Assay of Uracil Prototrophs and Rifampicin-Resistant Mutants From MK536 Derivatives

*Geobacillus kaustophilus* strains MK536_*p*__74_, MK536_*rsbV*_, MK536_*sigB*_, and MK536_*sigX*_ were precultured at 60°C in LB medium (20 mL). After the culture had reached proliferative phase (OD_600_ = 1) and stationary phase (plus four additional hours of incubation), the cells were collected and washed with sterile water (see above). An aliquot (10^5^–10^6^ cfu) was incubated at 65°C for 96 h on MU plates to obtain uracil prototrophs. The aliquot was also incubated on MM plates for 24 h to determine viable cell concentrations. The generation frequency of uracil prototrophs was defined as the ratio of generated uracil prototrophs to incubated viable cells (10^5^–10^6^ cfu). In addition, proliferative and stationary cells were spread on LB plates supplemented with rifampicin (10 mg/L) and incubated at 65°C for 24 h to obtain rifampicin-resistant mutants. The aliquots were also incubated on LB plates for 24 h to determine viable cell concentrations. The generation frequency of rifampicin-resistant mutants was defined as the ratio of generated rifampicin-resistant mutants to incubated viable cells (10^5^–10^8^ cfu).

### Statistical and Bioinformatic Analyses

Statistical significance was analyzed using unpaired Student’s *t*-tests (one-tailed) with Microsoft Excel 2016. The sequence comparison was performed using ClustalW^2^, and IS elements were predicted using ISsaga (Varani et al., 2011).

## Results

### Unexpected Generation of Uracil Prototrophs From *G. kaustophilus* MK536

**FIGURE 1:**
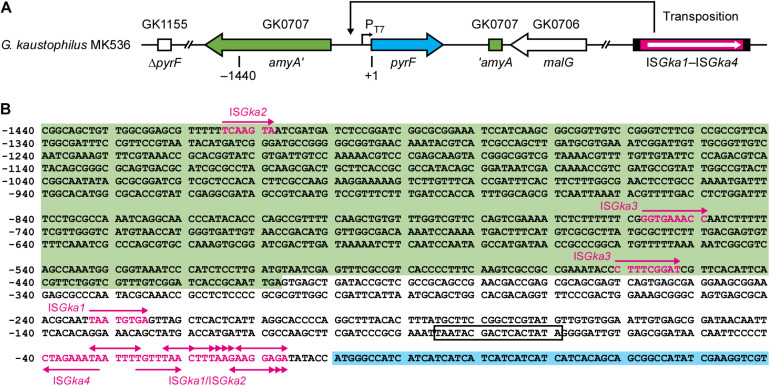
Organization of relevant genes in *Geobacillus kaustophilus* MK536. **(A)** *G. kaustophilus* MK536 lacks *pyrF* at GK1155 locus (Δ*pyrF*) but separately integrates *pyrF* (cyan) at GK0707 locus along with *amyA* disruption (green). The *pyrF* gene is under the control of T7 promoter (P_*T7*_), which is inactive in the absence of T7 RNA polymerase; therefore, *G. kaustophilus* MK536 is essentially auxotrophic for uracil. The chromosome contains insertion sequences (IS) termed IS*Gka1*, IS*Gka2*, IS*Gka3*, and IS*Gka4* (magenta) at three (GK0302, GK2085, and GK2942), two (GK0390 and GK1725), one (GK0169), and three loci (GK0015, GK2451, and GK3431), respectively. The numbers indicate positions where the initiation codon of *pyrF* is defined as +1. **(B)** Nucleotide sequence between the positions −1440 and +60. The *amyA* and *pyrF* regions are shown with green and cyan backgrounds, respectively, and P_*T7*_ is boxed. Uracil prototrophs were identified to carry IS elements in this region. Magenta arrows indicate the position and insertion direction of the IS elements, whereas magenta letters indicate sequences that resulted in direct repeats. Table 2 summarizes detailed information on the elements.

**FIGURE 2:**
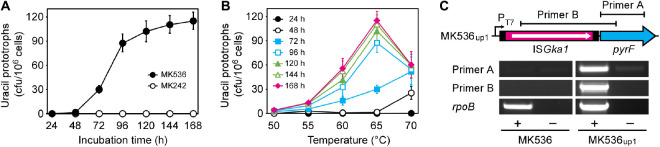
Generation of uracil prototrophs from *G. kaustophilus* MK536. **(A)** The time course of prototroph generation. *G. kaustophilus* strains MK536 (solid) and MK242 (hollow) were incubated at 65°C on MU plates to determine the generation frequencies of uracil prototrophs every 24 h. Data are presented as the mean ± standard error of cumulative numbers (*n* = 4–8). **(B)** The temperature profiles for prototroph generation. *G. kaustophilus* MK536 was incubated at 50–70°C on MU plates to determine the generation frequencies of uracil prototrophs every 24 h. Data are presented as the mean ± standard error of cumulative numbers (*n* = 4–8). **(C)** Transcription analysis of *pyrF* in *G. kaustophilus* strains MK536 and MK536_*up1*_. Total RNA was purified from cells that were cultured in MM medium and underwent reverse transcription (+). The products were used as templates for PCR to amplify the *pyrF* (0.6 kb) and IS*Gka1*-*pyrF* (1.4 kb) regions using primers A and B, respectively. The reaction without reverse transcriptase was used as negative control (–). A partial region (1.0 kb) of *rpoB* transcripts was detected as positive control.

*Geobacillus kaustophilus* MK536 lacks the *pyrF* gene essential for pyrimidine biosynthesis. Although the strain harbors intact *pyrF* under the P_*T*__7_ control (Figure 1), the gene is theoretically silent in the absence of T7RP that functions at elevated temperatures. Therefore, *G. kaustophilus* MK536 is auxotrophic for uracil; however, this strain unexpectedly produced uracil prototrophs when incubated on minimum medium without uracil (MU) at 65°C. The prototrophs appeared following incubation for >48 h (Figure 2A). When a prototroph (termed MK536_*up*__1_) was randomly selected and again incubated on MU plates, the majority of cells (>95%) formed colonies within 24 h as similar to those formed by the wild-type strain (HTA426). The prototrophy was stable and remained so throughout subculturing. The generation of uracil prototrophs was more rapid at 70°C but was most frequent at 65°C with prolonged incubation (Figure 2B). The optimal growth temperature of *G. kaustophilus* is 65°C (Suzuki, 2017); thus, uracil prototrophs were actively generated at temperatures where *G. kaustophilus* efficiently propagates. Prototrophs were not generated from a control strain lacking the P_*T*__7_-*pyrF* cassette (MK242).

### Uracil Prototrophs Carry IS Elements in the *pyrF* Upstream Region

For uracil prototrophy, it was hypothesized that P_*T*__7_ mutations were responsible for T7RP-independent *pyrF* expression because uracil prototrophs were not generated from *G. kaustophilus* MK242; thus, we sequenced upstream of *pyrF* in 49 prototrophs including *G. kaustophilus* MK536_*up*__1_. All the sequences lacked mutations but did contain different IS elements that encoded for DDE-type transposases (Figure 1). The IS elements were precisely flanked by DR that originated from the insertion site; therefore, it was probable that the event resulted from transposition but not from heterologous recombination. Among the 49 elements, 31 and 14 were completely identical with the IS elements at GK0302, GK2085, and GK2942 loci (termed IS*Gka1*; Supplementary Figure 1) and those at GK0390 and GK1725 loci (IS*Gka2*; Supplementary Figure 1), respectively. The IS*Gka2* sequence was highly homologous to the element at GK0778 locus but was distinguishable via a mutation. The other four sequences were completely identical to the IS element at GK0169 locus (IS*Gka3*; Supplementary Figure 2) or those at GK0015, GK2451, and GK3431 loci (IS*Gka4*; Supplementary Figure 3). The homologs of IS*Gka3* and IS*Gka4* were further identified at three (GK1006, GK1161, and GK1712) and five (GK0785, GK0875, GK1016, GK1720, and GKP33) loci, respectively. Based on sequence similarities, IS*Gka1* and IS*Gka2* were together classified to the IS*701* family, whereas IS*Gka3* and IS*Gka4* were classified to the IS*4* and IS*Lre2* families, respectively.

### Insertion Features of the IS Elements

Table 2 summarizes the classification, position, direction, and DR sequence of IS elements identified at the *pyrF* upstream site. The IS*Gka3* sequence was inserted in the parallel direction with the transposase and *pyrF* genes, whereas IS*Gka4* was inserted in the opposing direction. In agreement with observations for IS*4* and IS*Lre2* families (Siguier et al., 2015), IS*Gka3* and IS*Gka4* generated 9 bp of DR. The IS*Gka1* and IS*Gka2* sequences were inserted in both directions and occasionally at distant locations from the *pyrF* gene. Their transposition generated 4–9 bp of DR in disagreement with the observations of another set of IS*701* members, which are known to generate 4 or 5 bp of DR (Siguier et al., 2015). Hot spots were observed immediately upstream of the *pyrF* gene and were favored by IS*Gka1* and IS*Gka2*. Consensus sequences were not identified around insertion sites, although hot spots were abundant in adenine and thymine positions (Figure 1B).

**TABLE 2:** Insertion sequences identified at *pyrF* upstream in uracil prototrophs.

IS element	Insertion site	Direction	Direct repeat	Clone number
IS*Gka2*	−1415/−1409	Parallel	5′-TCAAGTA-3′	1
IS*Gka3*	−758/−750	Parallel	5′-GGTGAAACC-3′	1
IS*Gka3*	−461/−453	Parallel	5′-CTTTCGGAT-3′	1
IS*Gka1*	−233/−226	Parallel	5′-TAATGTGA-3′	1
IS*Gka4*	−40/−32	Opposite	5′-CTAGAAATA-3′	2
IS*Gka1*	−33/−27	Parallel	5′-TAATTTT-3′	2
IS*Gka1*	−33/−27	Opposite	5′-TAATTTT-3′	1
IS*Gka1*	−27/−21	Parallel	5′-TGTTTAA-3′	2
IS*Gka1*	−23/−20	Parallel	5′-TAAC-3′	1
IS*Gka1*	−23/−16	Parallel	5′-TAACTTTA-3′	1
IS*Gka1*	−23/−16	Opposite	5′-TAACTTTA-3′	1
IS*Gka1*	−23/−15	Parallel	5′-TAACTTTAA-3′	5
IS*Gka1*	−23/−15	Opposite	5′-TAACTTTAA-3′	7
IS*Gka2*	−23/−17	Parallel	5′-TAACTTT-3′	1
IS*Gka2*	−23/−16	Parallel	5′-TAACTTTA-3′	3
IS*Gka2*	−23/−15	Parallel	5′-TAACTTTAA-3′	2
IS*Gka2*	−23/−14	Parallel	ND	1
IS*Gka1*	−14/−9	Opposite	5′-GAAGGA-3′	1
IS*Gka1*	−14/−8	Parallel	5′-GAAGGAG-3′	1 (MK536_*up*__1_)
IS*Gka1*	−14/−7	Parallel	5′-GAAGGAGA-3′	3
IS*Gka1*	−14/−7	Opposite	5′-GAAGGAGA-3′	3
IS*Gka2*	−14/−7	Parallel	5′-GAAGGAGA-3′	5
IS*Gka2*	−14/−7	Opposite	5′-GAAGGAGA-3′	1
IS*Gka1*	−13/−7	Parallel	5′-AAGGAGA-3′	2

*Uracil prototrophs were generated from *G. kaustophilus* MK536, and the *pyrF* upstream in 49 prototrophs was sequenced to identify IS elements. The insertion site indicates possible sites where the element was inserted. The position corresponds to the upstream and downstream locations of the sequence that resulted in direct repeats. The position number is based on the original sequence (Figure 1B). The direction indicates that the element was inserted in the parallel or opposite direction for transposase and *pyrF* genes. An element was inserted between the −23 and −14 positions without direct repeats (ND). *G. kaustophilus* MK536_up__1_ carries IS*Gka1* with the parallel direction.*

### Transcription of *pyrF* in *G. kaustophilus* MK536_*up*__1_

Endpoint RT-PCR analysis showed that the *pyrF* gene was positively transcribed in MK536_*up*__1_ but not in MK536; there was a continuous transcript from IS*Gka1* upstream to *pyrF* (Figure 2C). Although faint bands were detected for MK536_*up*__1_ samples without RT via genomic DNA contamination, band signals were stronger for samples with RT. These observations suggested that *G. kaustophilus* MK536_*up*__1_ became prototrophic for uracil by *pyrF* expression via leaky and read-through transcription of the transposase gene and/or via active transcription from another promoter in IS*Gka1*.

### Genome-Wide IS Transposition in *G. kaustophilus* MK536_*up*__1_

**FIGURE 3:**
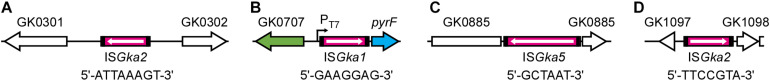
IS transposition in *G. kaustophilus* MK536_*up1*_. Genomic DNA was purified from *G. kaustophilus* MK536_*up1*_ and sequenced to identify genome-wide IS transposition. Magenta boxes indicate IS elements identified to achieve transposition in *G. kaustophilus* MK536_*up1*_ where arrows indicate the direction of transposase genes. Short sequences indicate direct repeats. IS*Gka2* was identified between transposase genes at GK0301 and GK0302 loci **(A)**, whereas IS*Gka1* was identified at the *pyrF* upstream site **(B)**. IS*Gka5* was identified in a hypothetical gene at GK0885 locus **(C)**. IS*Gka2* was also identified between hypothetical and RNA methyltransferase genes at GK1097 and GK1098 loci, respectively, **(D)**.

Next-generation sequencing of *G. kaustophilus* MK536_*up*__1_ provided 8 × 10^6^ reads and 169 contigs with 318 depth, which were compared with the complete genome sequence of *G. kaustophilus* HTA426 (Takami et al., 2004b). In addition to IS*Gka1* that transposed to the *pyrF* upstream region, IS*Gka2* and an IS element of the IS*1634* family were inserted at two and one loci, respectively, (Figure 3). The IS*Gka2* sequence was flanked by 7 or 8 bp of DR. The IS*1634* member was identical to the IS elements at GK0145 and GK3302 loci (termed IS*Gka5*; Supplementary Figure 4) and generated 6 bp of DR. The PCR assays confirmed that these elements were absent at the respective loci in *G. kaustophilus* MK536. Although another IS*1634* member was also identified at GK3299 locus, the element already existed in *G. kaustophilus* MK536; thus, it was likely that this transposed during construction of *G. kaustophilus* MK536 from *G. kaustophilus* HTA426. IS deletion was not identified in the genome sequence, which suggested that these elements achieved replicative transposition. Many mutations (134 single nucleotide variants, 50 deletions, and 81 insertions) were identified; however, it was unclear whether these mutations occurred during generation of MK536_*up*__1_ from MK536 or during construction of MK536 from HTA426.

### Southern Blot Analysis of IS*Gka1*/IS*Gka2* Transposition

**FIGURE 4:**
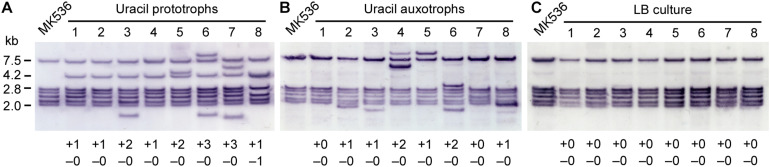
Southern blot analysis of IS*Gka1*/IS*Gka2* transposition. **(A,B)** *G. kaustophilus* MK536 (10^5^–10^6^ cfu) was incubated at 65°C on MU plates for 72 h. Grown colonies were used as uracil prototrophs **(A)**, whereas background cells were used as uracil auxotrophs **(B)**. Eight clones were randomly selected and analyzed by Southern blotting. The probe was designed to detect both IS*Gka1* and IS*Gka2*. The six bands for MK536 were attributable to fragments from the loci GK0778 (7.5 kb), GK2085 (2.8 kb), GK0302 (2.7 kb), GK0390 (2.4 kb), GK1725 (2.2 kb), and GK2942 (2.0 kb). At 4.2 kb, the *pyrF* upstream was detected carrying an IS element. The numbers at the bottom indicate an increase (+) and decrease (–) in bands. **(C)** *G. kaustophilus* MK536 was successively (seven times) cultured at 65°C in LB medium. Cells were purified on LB plates, and eight clones were analyzed by Southern blotting.

*Geobacillus kaustophilus* MK536 was incubated at 65°C for 72 h on MU plates to isolate uracil prototrophs, and eight clones were analyzed by Southern blotting that collectively detects IS*Gka1* and IS*Gka2* (Figure 4A). Six bands were present in the MK536 samples. The band lengths corresponded to the theoretical ones predicted from the sequences around the loci GK0778 (7.5 kb), GK2085 (2.8 kb), GK0302 (2.7 kb), GK0390 (2.4 kb), GK1725 (2.2 kb), and GK2942 (2.0 kb). The original bands remained present in the seven prototrophs; thus, IS*Gka1* and IS*Gka2* generally achieved replicative transposition that provided new information on the IS*701* family. One prototroph lost the band at GK0302 locus. Because an IS element uses either replicative or non-replicative mechanism, the loss seemed to result from a band shift via IS insertion around the locus, as observed for *G. kaustophilus* MK536_*up*__1_ (Figure 3A). A band was further shared at 4.2 kb, which was attributable to the *pyrF* upstream region that carries IS*Gka1* or IS*Gka2* on the basis of the theoretical length. Additional bands were also detected in four prototrophs. This supported IS transposition occurring in a genome-wide manner. In addition to uracil prototrophs, background cells that remained uracil auxotrophs were recovered from MU plates and analyzed by Southern blotting to show that the six clones increased the band signals at diverse lengths but not at 4.2 kb (Figure 4B). Notably, successive cultures under non-inhibitory growth conditions in LB medium resulted in undetectable IS*Gka1*/IS*Gka2* transposition (Figure 4C). These observations implied that IS*Gka1*/IS*Gka2* transposition extensively occurred in cells (>75%) on MU plates and that cells became uracil prototrophs when an IS element fortuitously transposed to the *pyrF* upstream region.

### Culture Conditions for IS*Gka1*/IS*Gka2* Transposition

*Geobacillus kaustophilus* MK536 was incubated in liquid MU at 65°C for 48 h and then grown on LB plates. Any subsequent colonies were classified as uracil prototrophs or auxotrophs, and eight clones were analyzed by Southern blotting to detect IS*Gka1* and IS*Gka2*. The signals were changed in five prototrophs and two auxotrophs (Table 3), which suggested that IS*Gka1*/IS*Gka2* transposition was enhanced not only on MU plates but also in liquid MU. The 4.2 kb signal was not detected in the prototrophs; however, PCR analysis confirmed that the *pyrF* upstream region had lengthened, suggesting that these clones might carry an IS element other than IS*Gka1* or IS*Gka2* at the *pyrF* upstream site. Similarly, MK536 cells were incubated in diverse media to prevent the cells from active propagation and then eight clones were recovered on LB plates to analyze IS*Gka1*/IS*Gka2* transposition. Incubation was performed for 72 h; however, the incubation time was shortened to 48 h when cells were not recovered. Table 3 summarizes the incubation conditions and the number of clones that achieved IS*Gka1*/IS*Gka2* transposition. Transposition was detected for cells that underwent growth inhibition by kanamycin, chloramphenicol, or carbon or nitrogen starvation. These observations suggested that IS transposition was enhanced under growth inhibitory conditions and may be regulated via a stress response pathway.

**TABLE 3:** IS*Gka1*/IS*Gka2* transposition under growth inhibitory conditions.

Stressor	Medium	Period	Transposition
Pyrimidine deficiency	MU (plate)	72 h	8/8 (uracil prototrophs); 6/8 (uracil auxotrophs)
	MU (liquid)	48 h	5/8 (uracil prototrophs); 2/8 (uracil auxotrophs)
Carbon starvation	MC (plate)	72 h	0/8
	MC (liquid)	72 h	1/8
Nitrogen starvation	MN (plate)	72 h	0/8
	MN (liquid)	72 h	1/8
Kanamycin	LB (plate)	48 h	2/8
	LB (liquid)	48 h	3/8
Chloramphenicol	LB (plate)	72 h	1/8
	LB (liquid)	72 h	1/8

**Geobacillus kaustophilus* MK536 was incubated at 65°C under growth inhibitory conditions (stressor and medium) for as long as possible (period). Resulting cells were purified on LB plates. Eight clones were analyzed for IS*Gka1*/IS*Gka2* transposition by Southern blotting. The numerator indicates how many clones achieved IS*Gka1*/IS*Gka2* transposition on the basis of different band patterns on Southern blots. When cells were incubated on MU plates, grown colonies were used as uracil prototrophs, whereas background cells were used as uracil auxotrophs. When cells were incubated in liquid MU, cells were colonized on LB plates and checked for uracil prototrophy to categorize into prototrophs and auxotrophs.*

### Expression of *sigX* Enhances Generation of Uracil Prototrophs From *G. kaustophilus* MK536

**FIGURE 5:**
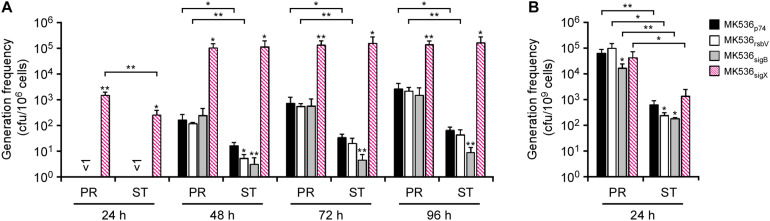
The effects of *rsbV*, *sigB*, and *sigX* expression on the generations of uracil prototrophs **(A)** and rifampicin-resistant mutants **(B)**. **(A)** *G. kaustophilus* strains MK536_*p74*_ (solid), MK536_*rsbV*_ (hollow), MK536_*sigB*_ (gray), and MK536_*sigX*_ (diagonal) were precultured until the proliferative (PR) and stationary (ST) phases were achieved and then incubated at 65°C on MU plates for 24–96 h to determine the generation frequencies of uracil prototrophs. **(B)** Proliferative and stationary cells were incubated at 65°C on LB plates supplemented with rifampicin (10 mg/L) for 24 h to determine the generation frequencies of rifampicin-resistant mutants. Data are presented as the mean ± standard error of cumulative numbers (*n* = 4). Statistical significance is shown between respective strains and MK536_*p74*_ (asterisks directly above the bars) and between proliferative and stationary cells (asterisks above the connection lines). Single and double asterisks indicate *P* < 0.15 and *P* < 0.05, respectively.

To see whether stress response regulators govern IS transposition in *G. kaustophilus*, we constructed MK536 derivatives that forcibly expressed *rsbV* (MK536_*rsvV*_), *sigB* (MK536_*sigB*_), and *sigX* (MK536_*sigX*_). The cells were precultured until they reached proliferative and stationary growth phases and then incubated at 65°C on MU plates to determine the generation frequency of uracil prototrophs (Figure 5A). As with *G. kaustophilus* MK536, the control strain (MK536_*p*__74_) generated prototrophs following incubation for >48 h. Similar observations were also made for MK536_*rsbV*_ and MK536_*sigB*_. In these strains, the generation frequency was higher in proliferative cells than in stationary cells. Although the stationary cells of MK536_*rsbV*_ and MK536_*sigB*_ exhibited lower frequency than did those of MK536_*p*__74_, the difference was not substantial. In contrast, MK536_*sigX*_ more rapidly and frequently generated uracil prototrophs than did the other strains. Moreover, the generation frequency was comparable between proliferative and stationary cells at >48 h. These observations suggested that IS transposition was enhanced via *sigX*-dependent stress responses and that the response was stronger in proliferative cells.

### Expression of *sigX* Has Negligible Effects on Rifampicin-Resistant Mutations

*Geobacillus* spp. apparently induce mutagenesis when proliferative cells are exposed to rifampicin and efficiently generate rifampicin-resistant cells via *rpoB* mutations (Suzuki et al., 2018). To see whether the mutagenesis depends on *sigX*-dependent stress responses, MK536 derivatives (MK536_*p*__74_, MK536_*rsbV*_, MK536_*sigB*_, and MK536_*sigX*_) were assessed by generation frequency assay of rifampicin-resistant mutants. Cells were precultured until they reached proliferative and stationary phases and incubated for 24 h at 65°C on LB plates supplemented with rifampicin. Grown colonies were counted to determine that the generation frequency of rifampicin-resistant mutants was higher in proliferative cells than in stationary cells for all of the strains, but substantial differences were not observed between the respective strains (Figure 5B). This suggested that the rifampicin-resistant mutations were independent from *sigX*-dependent stress responses.

## Discussion

Isothermal and transcription-based amplification of nucleic acids can be performed using T7RP, which is responsible for strong transcription from P_*T*__7_ (Niemz et al., 2011). Because amplification performance is potentially improved when conducted at higher temperatures, thermostable T7RP variants are of biotechnological importance (Boulain et al., 2013). This study was originally designed to generate mutant genes for thermostable T7RP variants in *G. kaustophilus* MK534_*T*__7__*RP*_. The strain is auxotrophic for uracil by the Δ*pyrF* genotype but carries *pyrF* under control of P_*T*__7_ (P_*T*__7_-*pyrF*); therefore, it could become prototrophic with functional expression of *T7RP* from pGKE75-*T7RP*. In addition, this strain lacks genes for DNA repair (*mutS*, *mutL*, *mutY*, *ung*, and *mfd*) and thereby could serve as an error-prone strain. We expected that *G. kaustophilus* MK534_*T*__7__*RP*_ would generate mutant genes for thermostable T7RP variants via culture and that such genes could be found in clones prototrophic for uracil at elevated temperatures. As expected, MK534_*T*__7__*RP*_ generated uracil prototrophs at 65°C; however, similar prototrophs were intrinsically generated from a control strain that lacked pGKE75-*T7RP* (MK536). Uracil prototrophs were not generated from another control strain without P_*T*__7_-*pyrF* (MK242); therefore, we assumed P_*T*__7_ mutations in uracil prototrophs and unexpectedly identified diverse IS elements at the *pyrF* upstream region (Figure 1). IS elements are known to cause neighboring gene expression from internal promoters or via formation of hybrid promoters (Zhang and Saier, 2016; Vandecraen et al., 2018). In fact, *pyrF* transcription was detected in MK536_*up*__1_ in contrast to MK536 (Figure 2C). In *G. kaustophilus* MK536_*up*__1_, a promoter upstream of the transposase gene apparently contributes to *pyrF* transcription. However, considering several prototrophs carried IS*Gka1*/IS*Gka2* or IS*Gka4* at the *pyrF* upstream region in the opposite direction (Table 2), they seem to harbor promoters with the opposite direction. Possible opposite promoters identified in IS*Gka1*/IS*Gka2* and IS*Gka4* are shown in Supplementary Figures 1 and 3, respectively.

*Geobacillus kaustophilus* HTA426 harbors a circular chromosome of 3.5 Mb and a large plasmid of 48 kb (Takami et al., 2004b), which have been predicted to carry 118 and 4 copies of possible IS elements, respectively (Table 4). The elements that achieved transposition include members of the families IS*4* (IS*Gka3*), IS*701* (IS*Gka1* and IS*Gka2*), IS*1634* (IS*Gka5*), and IS*Lre2* (IS*Gka4*). The copy number of these elements is 11, which suggest that >9% of the total elements (122 copies) are functional in *G. kaustophilus*. Among the elements, IS*Gka1* and IS*Gka2* were most frequently identified in the *pyrF* upstream region (Table 2). Their transposition was also more frequent in the MK536_*up*__1_ genome (Figure 3). Possible reasons for this include a higher copy number, replicative transposition, and abundant sequences to which IS*Gka1* and IS*Gka2* preferentially transpose. All the elements (IS*Gka1*–IS*Gka5*) transposed at elevated temperatures, indicating that these elements are thermophilic, which is in agreement with their distribution in thermophilic species of the family *Bacillaceae* (e.g., *Aeribacillus*, *Anoxybacillus*, *Bacillus*, and *Geobacillus* genera). Although several reports describe IS transposition in thermophiles (Sen and Oriel, 1990; Natarajan and Oriel, 1991; Xu et al., 1993; Schleper et al., 1994; Gregory and Dahlberg, 2008) or genetic modification using mesophilic mobile elements (Carr et al., 2015), no IS element has been hitherto demonstrated to preferentially function in thermophiles at elevated temperatures; thus, this study is the first to identify thermophilic IS elements. Mobile elements can be used for gene discovery and gene delivery (Picardeau, 2010; Narayanavari et al., 2017). Therefore, IS*Gka1*–IS*Gka5* have the potential to expand the genetic tools for thermophiles.

**TABLE 4:** IS elements predicted in *G. kaustophilus* HTA426.

Location	IS family	Subgroup	Transposase	Copy number	Note
Chromosome	IS*3*	IS*150*	DDE	3	
	IS*4*	IS*231*	DDE	1	
	IS*4*	IS*4Sa*	DDE	5	IS*Gka3*
	IS*5*	IS*5*	DDE	1	
	IS*6*	IS*6*	DDE	4	
	IS*21*		DDE	1	
	IS*66*	IS*Bst12*	DDE	5	
	IS*110*		DEDD	10	
	IS*200*/IS*605*		HUH/Y1	5	
	IS*200*/IS*605*	IS*1341*	HUH/Y1	7	
	IS*256*		DDE	7	
	IS*481*		DDE	8	
	IS*630*		DDE	4	
	IS*701*		DDE	7	IS*Gka1*/ IS*Gka2*
	IS*982*		DDE	6	
	IS*1634*		DDE	20	IS*Gka5*
	IS*L3*			10	
	IS*Lre2*		DDE	10	IS*Gka4*
pHTA426	IS*6*		DDE	1	
	IS*66*	IS*Bst12*	DDE	1	
	IS*Lre2*		DDE	2	

*The analysis was performed using ISsaga (Varani et al., 2011). Transposase indicates that DDE, DEDD, or HUH/Y1-type transposases are encoded in the family.*

Southern blot analysis suggested that IS*Gka1*/IS*Gka2* transposition frequently occurred in a genome-wide manner. Genome-wide transposition has been supported by the MK536_*up*__1_ genome, which carries additional IS elements at four loci (Figure 3). IS*Gka1*/IS*Gka2* transposition was detected not only in cells that became uracil prototrophs but also in background cells that remained uracil auxotrophs; however, transposition was not detected when cells were cultured under growth non-inhibitory conditions (Figure 4). These observations suggest that *G. kaustophilus* enhances IS transposition in response to pyrimidine deficiency and generated numerous mutants, including uracil prototrophs, where an element was fortunately transposed into the *pyrF* upstream region. The idea is consistent with growth of uracil prototrophs on MU plates after >48 h incubation. Given that prototrophs had randomly arisen during preculture, the colonies should have appeared within 24 h, as observed for *G. kaustophilus* strains HTA426 and MK536_*up*__1_. Uracil prototrophs identified in liquid MU supposedly carried another type of IS elements in the *pyrF* upstream region, which seems to perform transposition earlier than IS*Gka1* or IS*Gka2* under this condition because prototrophs generated earlier in the liquid culture rapidly grow and could become dominant throughout the subsequent phases. This observation also supports the IS transposition enhanced under pyrimidine deficient conditions.

Stress-induced transposition has been reported for several organisms by respective stressors (Zhang and Saier, 2016; Vandecraen et al., 2018; Lee et al., 2019, 2020). In *G. stearothermophilus* CU21, IS*4* and IS*21* members have been reported to achieve transposition during growth inhibition by chloramphenicol exposure (Xu et al., 1993). Notably, IS*Gka1*/IS*Gka2* transposition was detected in eight clones that underwent growth inhibition by antibiotic exposure or starvation; therefore, IS transposition may be enhanced by growth inhibition regardless of stressors. Although transposition was not observed under similar starvation conditions on solid media, this was attributed to nutrient contaminants in the agar enabling minimal growth under conditions of incomplete starvation. We note that transposition enhanced by growth inhibition helps organisms achieve genetic diversification that results in environmental adaptation exclusively during the period of growth inhibition; in parallel, transposition permitted by hosts is advantageous for IS elements in terms of their propagation. *Geobacillus* spp. harbor numerous IS elements in their genomes (Suzuki, 2018), which may reflect codependence relationships between *Geobacillus* spp. and IS elements for evolutionary diversification and stable persistence, respectively.

*Bacillus subtilis* 168 is a model bacterium that is phylogenetically related to *G. kaustophilus* (Suzuki, 2018). In *B. subtilis*, various stress responses are positively regulated by SigB where the function is repressed by RsbW (anti-SigB) via complex formation, whereas RsbW is released by RsbV (anti-SigB antagonist); therefore, SigB can be activated by RsbV (van Schaik and Abee, 2005). In *G. kaustophilus*, homologous genes for RsbV, RsbW, and SigB have been identified at the GK3422, GK3423, and GK3424 loci, respectively, (Takami et al., 2004b). Extra-cytoplasmic function (ECF) sigma factors are also known to positively regulate stress responses (van Schaik and Abee, 2005). Although *B. subtilis* employs multiple ECF sigma factors (e.g., SigM, SigV, SigW, SigX, SigY, and SigZ), only the homologs for *sigW* and *sigX* at their respective loci GK0150 and GK2254 have been identified in *G. kaustophilus* (Takami et al., 2004b).

Because IS transposition was apparently enhanced by growth inhibition, we focused on RsbV, SigB, SigW, and SigX as potential regulators that govern IS transposition in *G. kaustophilus* and constructed *rsbV* (MK536_*rsbV*_), *sigB* (MK536_*sigB*_), and *sigX* (MK536_*sigX*_) expressers. Despite repetitive trials, neither a *sigW* expressor nor most deletion mutants (Δ*sigW*, Δ*sigX*, Δ*rsbV*, or Δ*rsbW*) could be constructed. Notably, uracil prototrophs were rapidly and substantially generated from MK536_*sigX*_ in comparison with the other constructs (Figure 5A); therefore, it is possible that *G. kaustophilus* enhances IS transposition via *sigX*-dependent stress responses. This is the first observation suggesting that a sigma factor regulates IS transposition. In *B. subtilis*, SigX-dependent promoters share tgtAACnww and CGwCww consensus sequences at −35 and −10 regions, respectively, (Huang and Helmann, 1998). However, similar regions were not found upstream of transposase genes in IS*Gka1*–IS*Gka5* (Supplementary Figures 1–4), and therefore SigX seems to indirectly regulate IS transposition rather than to directly bind to IS elements. It is also noteworthy that SigX is involved in controlling several processes related to cell envelope modification in *B. subtilis* (Souza et al., 2014). Conceivably, *G. kaustophilus* may enhance IS transposition by sensing cell surface damage driven by growth inhibition via *sigX*-dependent stress responses.

Uracil prototrophs were equally generated from proliferative and stationary cells of MK536_*sigX*_ in contrast to other strains (Figure 5A). This profile suggests that *G. kaustophilus* enhances IS transposition under SigX regulation when proliferative cells are prevented from active propagation, thus potentially enabling immediate adaptation via genetic diversification. *Geobacillus* spp. can form robust endospores in the stationary phase (Suzuki, 2018); therefore, IS transposition may be enhanced as an adaptation strategy specific to the proliferative phase. We previously observed that exposure of *G. kaustophilus* to rifampicin apparently induced mutagenesis to produce rifampicin-resistant mutants and the induction was stronger in proliferative cells than in stationary cells (Suzuki et al., 2018). Although this manner implies that mutagenesis may be also governed by SigX in parallel to IS transposition, rifampicin-resistant mutants were comparably generated between MK536_*p*__74_ and MK536_*sigX*_ (Figure 5B); therefore, mutagenesis is not under the SigX control. Multiple mechanisms appear to be employed to generate genetic diversification in *G. kaustophilus* and potentially other *Geobacillus* spp. This characteristic may be a primary reason why *Geobacillus* spp. are highly adaptive organisms.

## Data Availability Statement

The datasets presented in this study can be found in online repositories. The names of the repository/repositories and accession number(s) can be found below: SRA database PRJNA699136.

## Author Contributions

HS had conceived the experiment plan, supervised the experiment process, and wrote the original manuscript. JK examined *T7RP* mutations and identified IS transposition. MT and MO analyzed the transposition frequency and insertion sites of IS elements. TT performed the Southern blot and mutation assay. MO performed the transcriptome and genome analyses. TO had supervised the experiment process. All authors contributed to the article and approved the submitted version.

## Conflict of Interest

The authors declare that the research was conducted in the absence of any commercial or financial relationships that could be construed as a potential conflict of interest.

## Footnotes

1 https://blast.ncbi.nlm.nih.gov

2 http://www.ddbj.nig.ac.jp
