# Supplementary material for: Frequent Transposition of Multiple Insertion Sequences in Geobacillus kaustophilus HTA426
Source: Front Microbiol. 2021 Mar 24;12:650461. doi: 10.3389/fmicb.2021.650461 (PMC8024623; doi:10.3389/fmicb.2021.650461)
Supplement: Supplementary file 1 [file Data_Sheet_1.PDF]

**Supplementary Table 1.** Primers used in this study

| Primer | Sequence (5′–3′) |
|---|---|
| amyA$_{200F}$ | GCCGCATGCGACAAACGACCAGAACGTG |
| amyA$_{400F}$ | CCAACCATGTCGGTTACAATCACC |
| amyA$_{400R}$ | GACATGGTTGGCGACAAAATCC |
| amyA$_{800R}$ | CGACAAAACCGTCGATGCCGTATTG |
| amyA$_{1300R}$ | TCCAATAAAAAGCCGCGCAGTTCC |
| gk704$_{-250F}$ | GCCGCATGCAAGCTTTTTCTTTTTCCTCCTTTGTTATC |
| is25F | AGAAACCTCTCTAGTAGCGG |
| is25R | CACGGCATGGTGAATACTTG |
| is28F | ACCGGAGGAAGAACTTATGG |
| is28R | ATGTCCCGGAAATGACTCTC |
| is72F | CCGATTTCAAGCCTAGACTC |
| is72R | GTCTGACTGATCCTCTGTTG |
| is87F | TTTATACGGTATGCCGACCC |
| is87R | AGTGTCTCTTAACCCTTGCC |
| is701$_{250F}$ | CTTGACCACCAAGGGATTCTC |
| is701$_{800R}$ | GAATCCGGTTCGTCTTGAGCATC |
| pyrF$_{0F}$ | GCCGCATGCACACGCCGTTCATTGTC |
| pyrF$_{20R}$ | CTCGGATCCTCAAGCGCGACAATGAACGG |
| pyrF$_{200R}$ | GTTTCACCGTATTCGGAATG |
| pyrF$_{600R}$ | TCATCAGCAAAGCGGATACCC |
| pyrF$_{TR}$ | GCCGGATCCTTAAGTGGGGGTAGTTGACTCTC |
| rpoB$_{2800F}$ | GGGTTATTTCCCGCATTTTG |
| rpoB$_{3800R}$ | TCCGATAGTTGATTGTCTCC |
| rsbV$_{0F}$ | GACAGGAGGGGGATTATGGATCAGAAACAATTCC |
| rsbV$_{0R}$ | GGAATTGTTTCTGATCCATAATCCCCCTCCTGTC |
| rsbV$_{TR}$ | GCCGGATCCTCATGATTGGCCTCCGATCCTTTC |
| t7$_{-250F}$ | GCCAAGCTTCGATCCCGCGAAATTAATAC |
| t7$_{0R}$ | GGCGCATGCGACGACCTTCGATATGGCCGCTGCTG |
| t7RP$_{0F}$ | GCCGCATGCAGACGATTAACATCGCTAAG |
| t7RP$_{TR}$ | GCCGGATCCTTACGCGAACGCGAAGTCCGAC |
| sigB$_{0F}$ | GACAGGAGGGGGATTATGACTGTATATCCCCCTC |
| sigB$_{0R}$ | GAGGGGGATATACAGTCATAATCCCCCTCCTGTC |
| sigB$_{TR}$ | GCCGGATCCTTTATGGCGAATAGGCGG |
| sigX$_{0F}$ | GACAGGAGGGGGATTATGGACCCCGTCTTTGAAC |
| sigX$_{0R}$ | GTTCAAAGACGGGGTCCATAATCCCCCTCCTGTC |
| sigX$_{TR}$ | GCCGGATCCTCATTCCAATTGAGCTTTTTC |

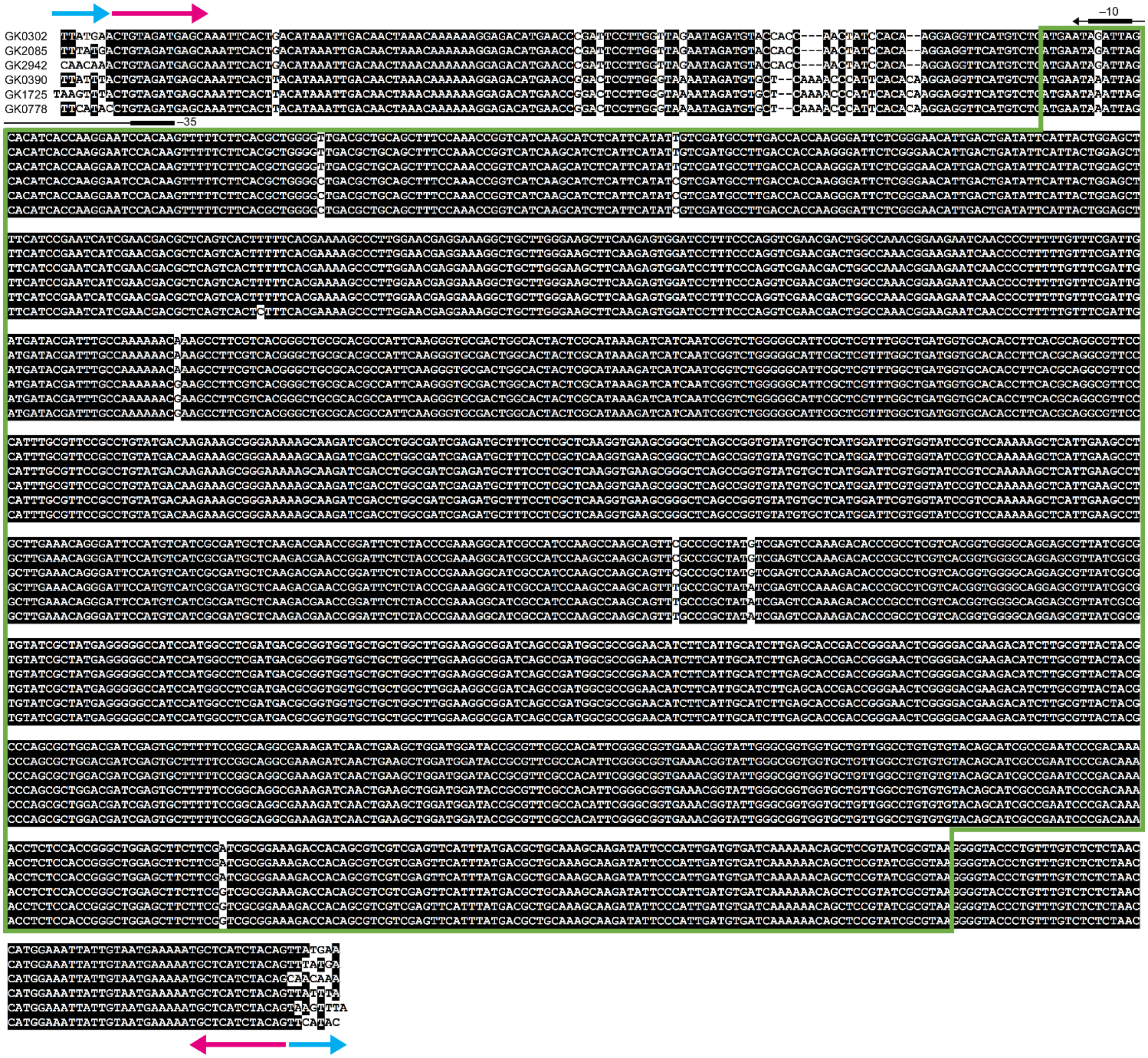


**Supplementary Figure 1.** Nucleotide sequences of IS*Gka1* and IS*Gka2*

Sequences are shown with locus tags of transposase genes (green box). Magenta and cyan arrows indicate inverted and direct repeats, respectively. The elements are identical among GK0302, GK2085, and GK2942 loci (IS*Gka1*) and between GK0390 and GK1725 loci (IS*Gka2*). Although IS*Gka2* and the element at GK0778 locus are almost identical, these elements can be distinguished with a mutation. The −10 and −35 regions indicate a potential opposite promoter.

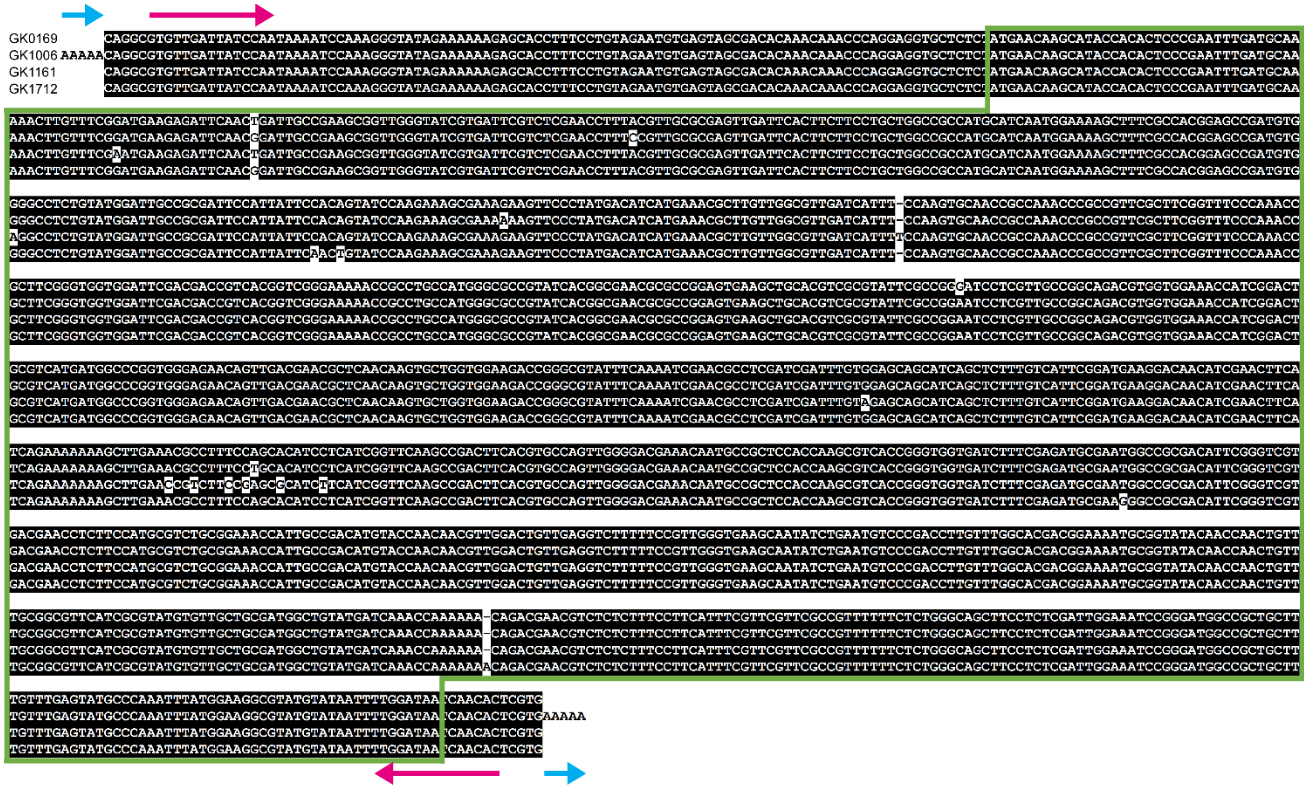

**Supplementary Figure 2.** Nucleotide sequences of IS*Gka3* and its homologs

The sequences are shown with locus tags of transposase genes (green box). The element at GK0169 locus corresponds to IS*Gka3*. Magenta and cyan arrows indicate inverted and direct repeats, respectively. All the elements are distinguishable with mutations. The element at GK1161 locus has a frame-shift mutation in the transposase gene.

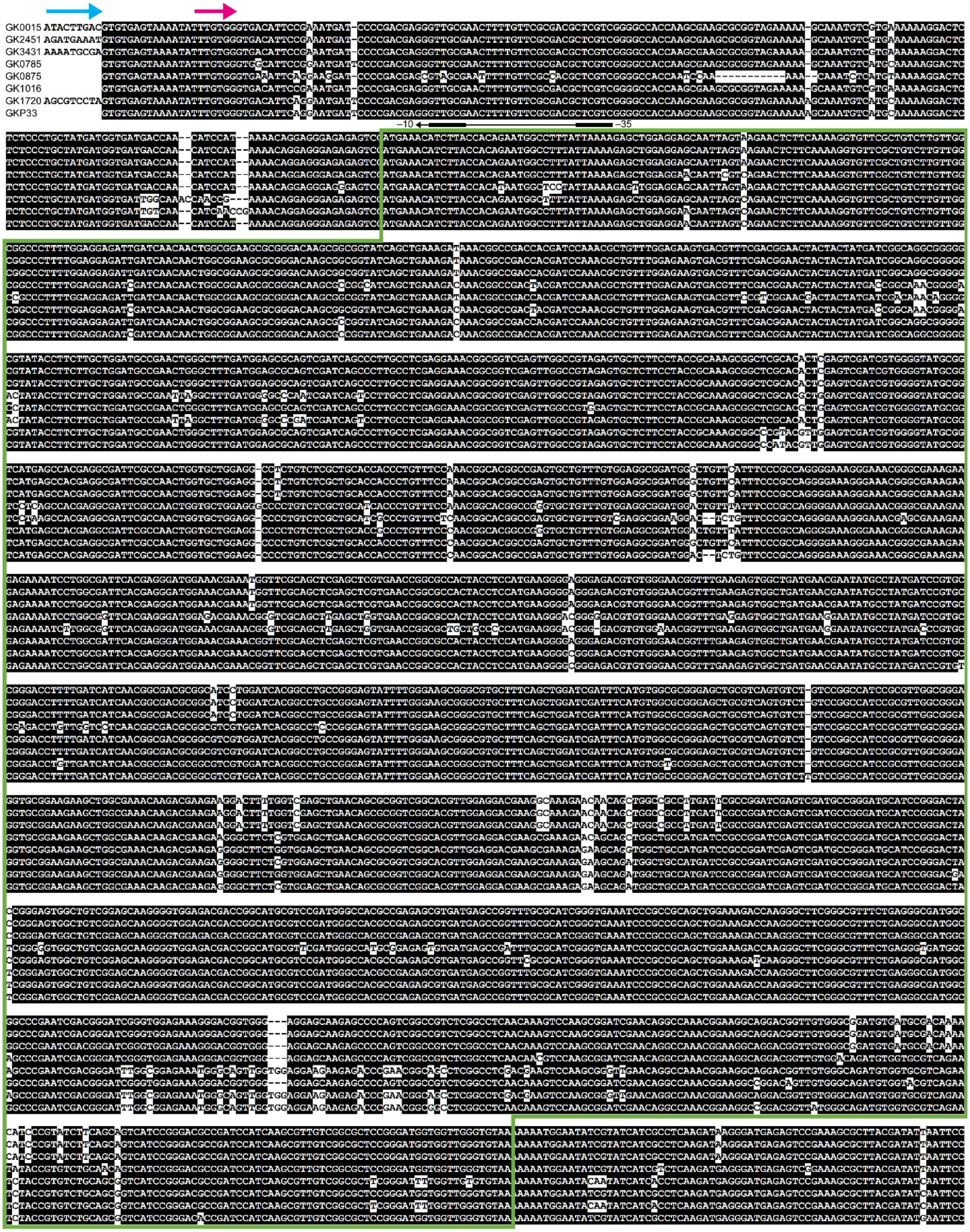
GK0015 ATACTTGAC
GK2451 AGATGAAAT
GK3431 AAAATGCGA
GK0785
GK0875
GK1016
GK1720 AGCGTCCTA
GKP33
–10
–35

Figure 3 continued

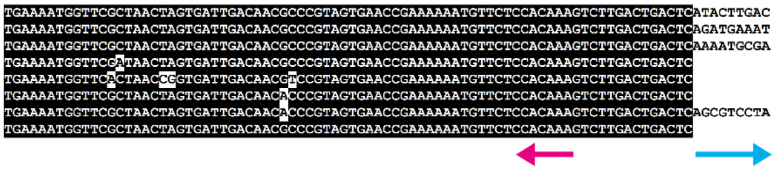


**Supplementary Figure 3.** Nucleotide sequences of IS*Gka4* and its homologs

The sequences are shown with locus tags of transposase genes (green box). Magenta and cyan arrows indicate inverted and direct repeats, respectively. The elements are identical among GK0015, GK2451, and GK3431 loci (IS*Gka4*). The elements at GK0785, GK0875, and GKP33 loci have frame-shift mutations in the transposase genes. The −10 and −35 regions indicate a potential opposite promoter.

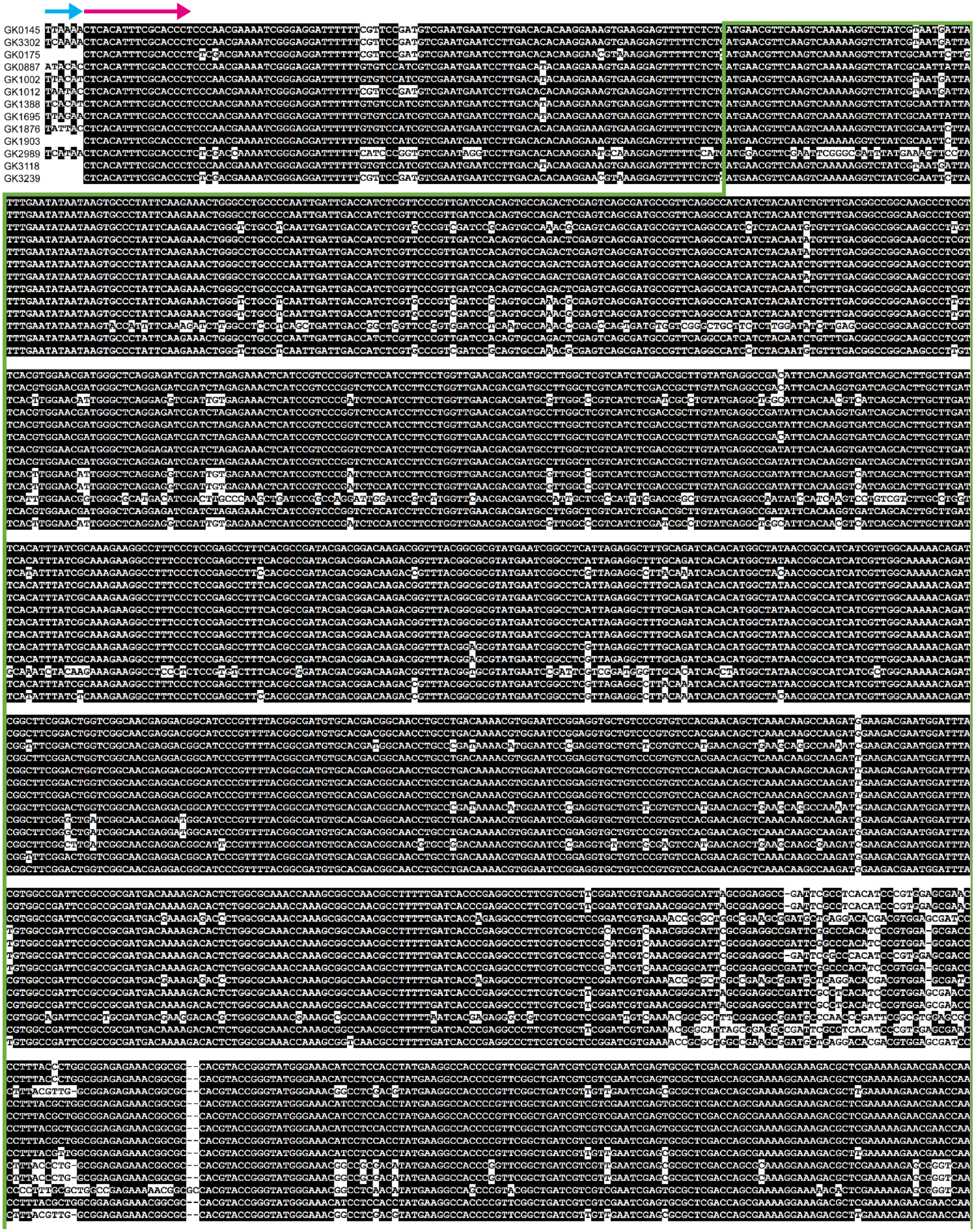
GK0145
GK3302
GK0175
GK0887
GK1002
GK1012
GK1388
GK1695
GK1876
GK1903
GK2989
GK3118
GK3239

Figure 4 continued

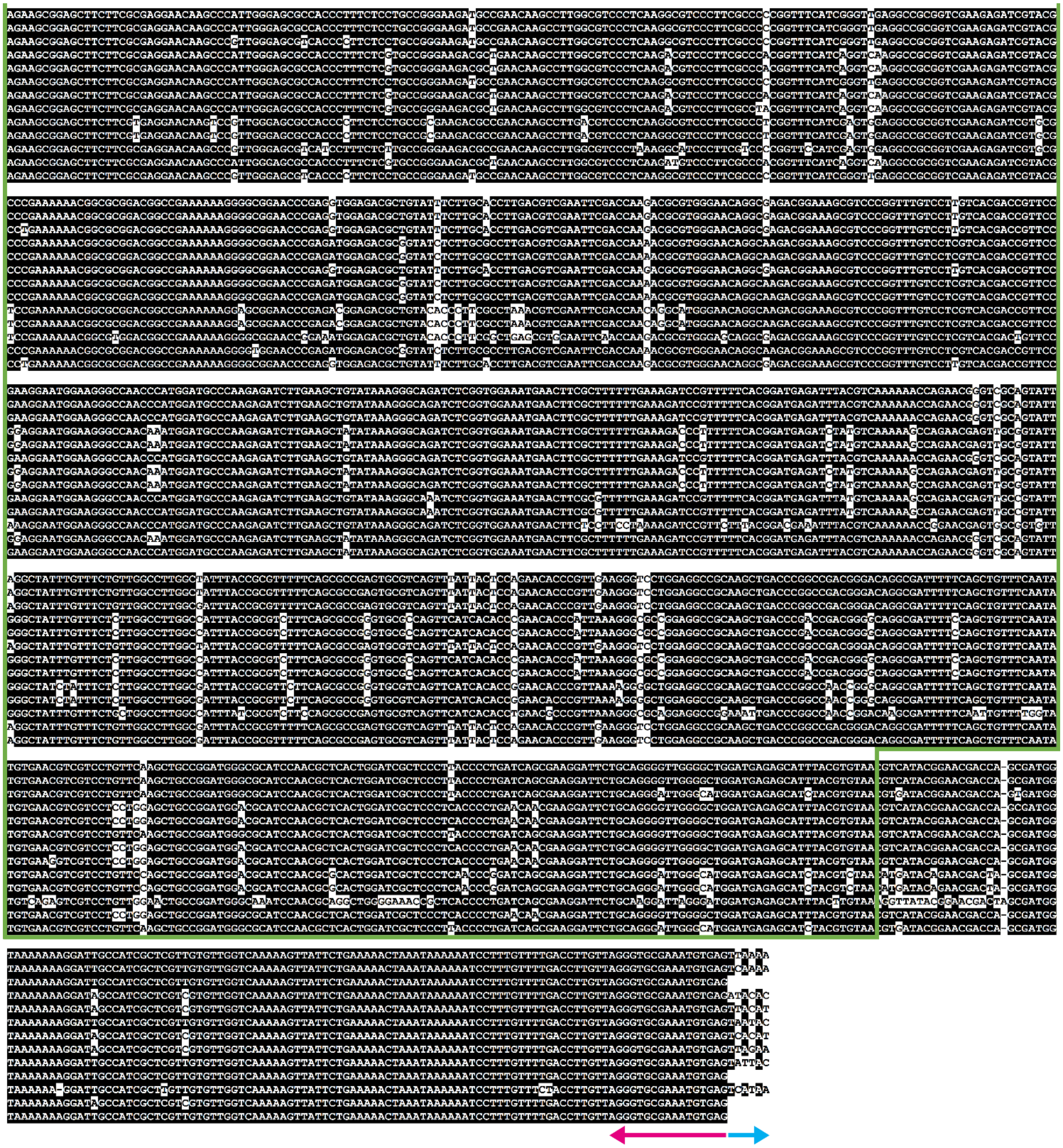

**Supplementary Figure 4.** Nucleotide sequences of IS*Gka5* and its homologs

Sequences are shown with locus tags of transposase genes (green box). Magenta and cyan arrows indicate inverted and direct repeats, respectively. The element at GK0145 and GK3302 loci corresponds to IS*Gka5*, which can be distinguished from the other 11 elements with mutations.
